# *β*-Arrestin1/miR-326 Transcription Unit Is Epigenetically Regulated in Neural Stem Cells Where It Controls Stemness and Growth Arrest

**DOI:** 10.1155/2017/5274171

**Published:** 2017-02-12

**Authors:** Agnese Po, Federica Begalli, Luana Abballe, Vincenzo Alfano, Zein Mersini Besharat, Giuseppina Catanzaro, Alessandra Vacca, Maddalena Napolitano, Marco Tafani, Felice Giangaspero, Franco Locatelli, Elisabetta Ferretti, Evelina Miele

**Affiliations:** ^1^Department of Molecular Medicine, Sapienza University of Rome, Rome, Italy; ^2^Department of Experimental Medicine, Sapienza University of Rome, Rome, Italy; ^3^Department of Radiological, Oncological and Anatomopathological Sciences, Sapienza University of Rome, Rome, Italy; ^4^IRCCS Neuromed, Pozzilli, Italy; ^5^Department of Hematology/Oncology and Stem Cell Transplantation, Bambino Gesù Children's Hospital, IRCCS, Rome, Italy; ^6^Center for Life NanoScience@Sapienza, Istituto Italiano di Tecnologia, Rome, Italy

## Abstract

Cell development is regulated by a complex network of mRNA-encoded proteins and microRNAs, all funnelling onto the modulation of self-renewal or differentiation genes. How intragenic microRNAs and their host genes are transcriptionally coregulated and their functional relationships for the control of neural stem cells (NSCs) are poorly understood. We propose here the intragenic miR-326 and its host gene *β*-arrestin1 as novel players whose epigenetic silencing maintains stemness in normal cerebellar stem cells. Such a regulation is mediated by CpG islands methylation of the common promoter. Epigenetic derepression of *β*-arrestin1/miR-326 by differentiation signals or demethylating agents leads to suppression of stemness features and cell growth and promotes cell differentiation. *β*-Arrestin1 inhibits cell proliferation by enhancing the nuclear expression of the cyclin-dependent kinase inhibitor p27. Therefore, we propose a new mechanism for the control of cerebellar NSCs where a coordinated epigenetic mechanism finely regulates *β*-arrestin1/miR-326 expression and consequently NSCs stemness and cell growth.

## 1. Introduction

Neural stem cells (NSCs) are believed to foster a hierarchical developmental program in which self-renewal and pluri/multipotency are responsible for the expansion and/or the maintenance of an uncommitted cell population pool. NSCs are ready to undergo the cascade of lineage restriction and subsequent terminal differentiation under the action of regulatory morphogenic cues [1]. The early postnatal murine cerebellum contains multipotent NSCs, which can be isolated and expanded in vitro. NSCs maintain their undifferentiated phenotype under mitogenic signals and are able to differentiate into the different kind of neurons [2].

Recent evidences have highlighted the crucial role of microRNAs (miRNAs) in conferring neural cell identities during neural induction, neuronal differentiation, and subtype specification [3]. MiRNAs are widespread throughout the genome, where they can be found in either intergenic or intragenic (especially intronic) regions [4]. Transcription of both intergenic and intragenic miRNAs may be regulated by their own promoters, whether some intragenic miRNAs share promoters with their host genes that generate pre-miRNA and mRNA, both arising from the same transcript [5, 6]. The consequent spatial and temporal coexpression implies a functional relationship between the intragenic miRNA and its host gene.

We previously identified miR-326 as a miRNA that is required to differentiate cerebellar granule cell progenitors (GCPs) to mature granule cells [7]. This miRNA is embedded within the first intron of *β-arrestin1 (βarr-1)* gene and shares with its host gene the same regulatory sequences [8, 9]. Since stem cell commitment to GCPs is a crucial event in cerebellar development [2, 10] we raised the question of miR-326 expression and regulation in cerebellar NSC. We report here that miR-326 expression is further downregulated in NSC prior to progenitor commitment, along with the downregulation of its host gene. Therefore, we investigated the expression, functions, and regulation of* miR-326/βarr-1* transcription unit in NSCs.

## 2. Materials and Methods

Unless otherwise indicated, media and supplements were purchased from Gibco-Invitrogen (Carlsbad, CA), chemicals were purchased from Sigma-Aldrich (St. Louis, MO), and commercial products were used according to the manufacturers' instructions/protocols.

### 2.1. Cell Culture

#### 2.1.1. Neural Stem Cells Culture

Mouse cerebella were obtained from postnatal 4-day-old wild-type BL6 mice (Charles River) with the approval of Institutional Review Board [8, 9]. Briefly, tissues were collected in HBSS supplemented with 0.5% glucose and penicillin-streptomycin, grossly triturated with serological pipette and treated with DNAse I to a final concentration of 0.04% for 20 minutes. Cells were mechanically dissociated using pipettes of decreasing bore size to obtain a single-cell suspension. Neural stem cells (NSCs) were derived and enriched through selective medium (SM): DMEM/F12 supplemented with 0.6% glucose, 25 mg/mL insulin, 60 mg/mL N-acetyl-L-cysteine, 2 mg/mL heparin, 20 ng/mL EGF, 20 ng/mL bFGF (Peprotech, Rocky Hill, NJ), penicillin-streptomycin, and B27 supplement without vitamin A. For clonogenicity assay cells were plated at clonal density (1-2 cells/mm^2^) into 96-well plate. To induce differentiation, NSCs were plated onto D-poly-lysine-coated dishes in differentiation medium (DFM: DMEM/F12 with N2 supplement and 2 mg/mL heparin, 0.6% glucose, and 60 mg/mL N-acetyl-L-cysteine, containing 1% Calf Serum and PDGF 10 ng/mL (Sigma, P3076) or RA 2 mM (Sigma, R2625), for 48 hours).

#### 2.1.2. Overexpression Studies

Amaxa nucleofector (Lonza) was used to transfect plasmids according to manufacturer's procedure. pcDNA3 *β*-arrestin1 HA was a gift from Robert Lefkowitz (Addgene plasmid # 14693): [11]; miR-326 vector and its negative control were purchased from GeneCopoeia (MmiR3333-MR01).

#### 2.1.3. Knockdown Experiments

Silencing of *β*-arrestin1 was performed using ON-TARGETplus SMARTpool (L40976-00-005) from Thermo Scientific, after testing each single siRNA of the pool, alone or in combination, for its specificity to avoid OFF-target effects.

#### 2.1.4. Cell Proliferation

Cell proliferation was evaluated by BrdU incorporation as previously described [2]. More in detail, after 24-hour pulse with BrdU, NSCs were plated on poly-lysine-coated Lab-Tek chamber slides (coverslips) and allowed to adhere for 3 hours. Cells were fixed with 4% paraformaldehyde, permeabilized with 0.1% Triton X-100, and BrdU detection (Roche) was performed. Nuclei were counterstained with the Hoechst reagent. Cells were counted in triplicate and the number of BrdU-positive nuclei was annotated.

NSC growth was measured by MTT assay (Promega) according to manufacturer's instructions. Each sample was measured in triplicate and repeated at least three times.

For methylation analysis, NSCs were treated with 10 *μ*M 5-azacytidine (5-AZA) for four days.

### 2.2. RNA Isolation and Gene Expression Analysis

Total RNA was purified and reverse transcribed as previously described [12]. Quantitative RT-PCR (qRT-PCR) analysis was performed using the ABI Prism 7900HT Sequence Detection System (Thermo Scientific), using best coverage TaqMan gene expression assays, specific for each analyzed mRNA. Each amplification was performed in triplicate and the average of the three threshold cycles was used to calculate the amount of transcripts (Thermo Scientific). mRNA quantification was expressed, in arbitrary units, as previously described [13]. mRNA quantification was expressed, in arbitrary units, as the ratio of the sample quantity to the calibrator or to the mean values of control samples. All values were normalized to three endogenous controls, *β*-actin, *β*2-microglobulin, and Hprt. miRNA expression levels were evaluated on RNA samples using specific stem-loop primers to achieve retrotranscription (Thermo Scientific) and was followed by a quantitative RT-PCR using miRNA-specific TaqMan probes (Thermo Scientific) in a ABI Prism 7900HT Sequence Detection System (Thermo Scientific). miRNA expression levels were normalized to RNAU6B [14].

### 2.3. Western Blot Assay

Cells were lysed using RIPA buffer (Tris-HCl pH 7.6 50 mM, deoxycholic acid sodium salt 0.5%, NaCl 140 mM, NP40 1%, EDTA 5 mM, NaF 100 mM, sodium pyrophosphate 2 mM, and protease inhibitors). Lysates were separated on 8% acrylamide gel and immunoblotted using standard procedures. The following antibodies were used: anti-*β*-arrestin1 K-16 (sc-8182; Santa Cruz Biotechnology, CA), anti-mouse Nanog (Cosmo Bio Co, Japan), anti-mouse *β*III-tubulin (MAB 1637 Millipore), anti-actin I-19 (sc-1616; Santa Cruz Biotechnology, CA), anti-GAPDH (ab8245; Abcam), anti-Sp1 1C6 (sc-420X; Santa Cruz Biotechnology, CA), anti-p27 (F-8) (sc-1641; Santa Cruz Biotechnology), anti-Sox2 (MAB4343 Millipore Billerica, MA), anti-Oct4 (ab19857; Abcam), anti-mouse *β*III Tubulin (MAB 1637 Millipore Billerica, MA), anti-GFAP MAB360 (Millipore Billerica, MA), and anti-PARP p85 fragment (G7342 Promega) (anti-parp-C). HRP-conjugated secondary antibodies (Santa Cruz Biotechnology, CA) were used in combination with enhanced chemiluminescence (ECL Amersham, Amersham, UK). For nucleus/cytoplasmic fractionation, cells were lysed on ice with buffer containing 20 mM Hepes, pH 7.4, 20% glycerol, 50 mM KCl, 1 mM EDTA, 5% NP 40, and protease inhibitors. After centrifugation the cytoplasmic fractions were collected (supernatant). The pelleted nuclear fraction was lysed with buffer containing 20 mM Hepes, pH 7.4, 25% glycerol, 400 mM NaCl, 1 mM EDTA, 1 mM EGTA, and protease inhibitors.

### 2.4. In Situ Hybridization and Immunofluorescence Assay

All reagents used prior to probe hybridization were prepared with DEPC-treated water (diethyl pyrocarbonate, Sigma D5758) to prevent RNAse contamination.

Cells were fixed with 4% PFA for 10 minutes at room temperature. After 3 washes with PBS, cells were incubated for 2 minutes with 10 *μ*g/mL proteinase K (Sigma P2308) at 37°C.

Acetylation was then performed to enhance signal to background ratio, using 1.2% triethanolamine (Sigma 90279), 0.0018 N HCl, and 0.25% acetic anhydride (Sigma A6404) with constant stirring for 10 minutes at room temperature. Cells were washed 3 times with PBS and prehybridization was performed using 50% formamide (Sigma F9037), 5XSSC buffer (FLUKA cat. S6639-1L), 0,1% Tween-20, 9,2 mM citric acid (Sigma C1909), 50 *μ*g/mL heparin (Sigma H4784), and 500 *μ*g/mL yeast RNA (Sigma R6750) for 3 hours at 62°C. Hybridization probe (double DIG-labeled, Exiqon) was diluted in hybridization buffer at a final concentration of 25 nM, denaturated at 85°C for 5 minutes, cooled on ice, and then incubated on cells at 62°C for 16 hours.

Samples were incubated with 0.1x SSC buffer for 3 hours at 67°C and washed 3 times with 0.1 M Tris-HCl pH 75 and 0.15 M NaCl. Nonspecific antibody binding was performed with 0.5% blocking reagent (Roche 11096176001), 5% Sheep Serum (Sigma S3772), in 50 mM Tris-HCl pH 7.5, and 5 mM EDTA for 2 hours at room temperature in a humidified chamber. Fluorescein conjugated anti-DIG was incubated at the dilution of 1 : 200 in blocking buffer for 16 hours at 4°C. After antibody incubation, samples were washed 3 times with 0.1 M Tris-HCl pH 75 and 0.15 M NaCl and coverslips were mounted using fluorescent mounting (DAKO S3023).

Immunofluorescence was performed as previously described [13]. More in detail, NSCs were cultured in Lab-Tek chamber slides fixed in 4% paraformaldehyde for 20 min at room temperature, permeabilized with 0.1% Triton X-100 cells, and incubated in blocking buffer (PBS with 1% BSA) for 30 min and then with anti-HA (sc-7392 Santa Cruz) overnight in blocking solution at 4°C. 488-conjugated anti-rabbit secondary antibody was purchased from Molecular Probes (Invitrogen). Nuclei were counterstained with Hoechst (H6024 Sigma). At least 300 nuclei were counted in triplicate. Fluorescence was visualized and images were acquired with Carl Zeiss microscope (Axio Observer Z1) using ApoTome technology and AxioVision Digital Image Processing Software.

### 2.5. Analysis of CpG Islands Methylation

miR-326/*β*arr-1 regulatory region was retrieved by Rulai database (http://rulai.cshl.edu/TRED/).

CpG islands were identified in the regulatory regions of* miR-326/βarr-1* by using database of CpG islands and Analytical Tool (DBCAT) software.

The primers for methylation specific PCR were designed by using Methyl Primer Express Software v1.0, Thermo Fisher Scientific. Primers were first tested and validated using mouse Universal Methylated Mouse DNA Standard (Zymo Research).

#### 2.5.1. Bisulphite Treatment and PCR Amplification

DNA extraction was performed using the Qiamp DNA mini kit (Qiagen). The obtained DNA was quantified using Nanodrop spectrophotometer (Thermo Scientific) and treated with EpiTect Bisulfite kit (Qiagen). The converted DNA was used to PCR-amplify the *β*arr-1 regulatory region with the following primers:

  MeFw: TTTTTATTTTTTGGGCGCGTATAC
  MeRw: GTCCAAACTAAAAAATCCCCGAC
  UnFw: TTTTTATTTTTTGGGTGTGTATATGT
  UnRw: CATCCAAACTAAAAAATCCCCAAC

As positive control we used mouse Universal Methylated Mouse DNA Standard (Zymo Research).

### 2.6. Statistical Analysis

Statistical analysis of cellular experimental triplicates was performed using StatView 4.1 software (Abacus Concepts, Berkeley, CA). Statistical differences were analyzed by Mann–Whitney *U* test for nonparametric values and a *p* value of ≤0.05 was considered significant. The results are expressed as mean ± SD from at least three experiments.

## 3. Results

### 3.1. miR-326 and *βarr-1* Expression Inversely Correlates with Stemness

**Figure 1:**
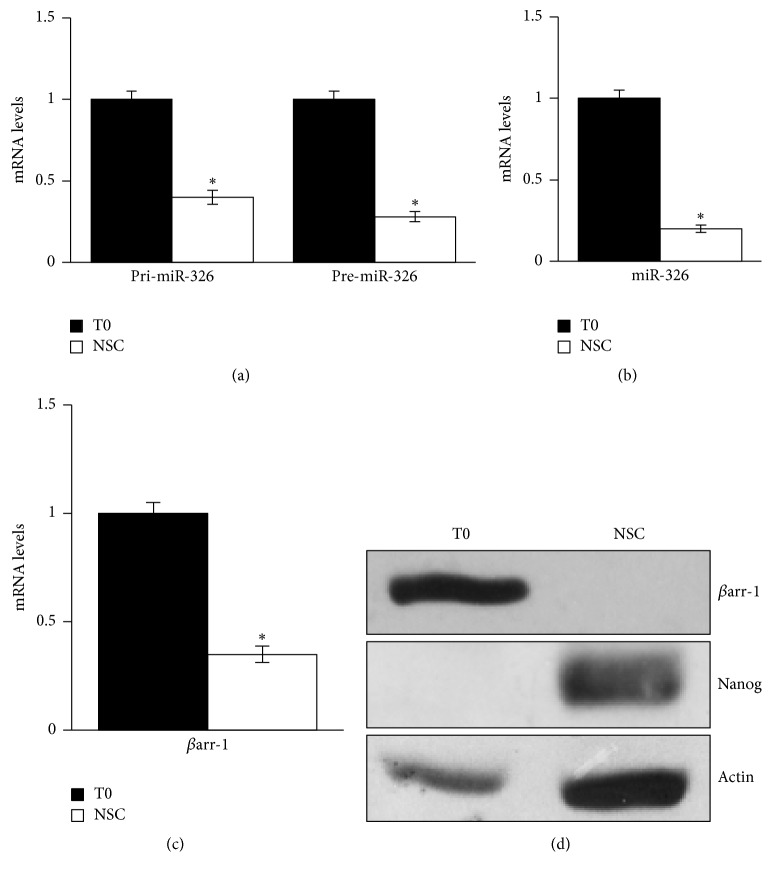
miR-326 and *β*arr-1 are downregulated in cerebellar NSCs compared to the bulk cerebellar population. (a) mRNA expression levels of the primary (pri-miR-326) and precursor (pre-miR-326) of miR-326 in NSCs compared to bulk cells (T0). (b) Mature miR-326 levels in NSCs compared to bulk cells (T0). (c) *β*arr-1 mRNA levels in NSCs compared to bulk cells (T0). (d) Western Blot (WB) analysis of endogenous *β*arr-1 and Nanog in NSCs compared to bulk cells (T0). Actin as loading control (LC). (a)–(d) data are means ± SD from 3 independent experiments. ^*∗*^*p* < 0.05.

First we evaluated expression levels of miR-326 and of its host gene *βarr-1* in NSCs (Figures 1(a)–1(d)). NSCs displayed, as compared to the bulk preneurosphere cell population (T0), very low levels of both mature and precursor miR-326 forms and of *βarr-1* (Figures 1(a)–1(d)), together with an enhanced expression of the Nanog stemness marker (Figure 1(d)).

**Figure 2:**
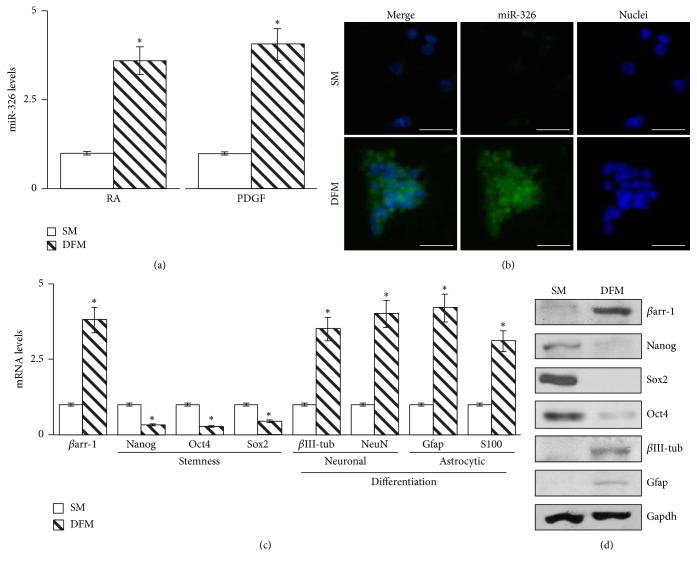
miR-326 and *β*arr-1 are upregulated in differentiated NSCs. (a) miR-326 levels evaluated in NSC exposed to differentiation stimuli (DFM) with retinoic acid (RA) or platelet-derived growth factor (PDGF) for 48 hours. Data are means ± SD from 3 independent experiments. ^*∗*^*p* < 0.05. (b) Fluorescent in situ hybridization (ISH) staining of miR-326 of NSCs grown in stemness conditions (SM) and after RA-induced differentiation (DFM) for 48 hours. Nuclei are counterstained with Hoechst. Scale bar: 10 *μ*m. Representative ISH images from 4 independent experiments. (c) mRNA expression levels of *β*arr-1 along with stemness and differentiation markers of NSC in SM and after differentiation (DFM). (d) Western blot (WB) analysis of endogenous *β*arr-1 and markers of stemness (Nanog, Sox2, and Oct4) and differentiation (*β*III-tub and Gfap) of NSCs in SM and after differentiation (DFM). Gapdh as loading control (LC). ((c) and (d)) Data are means ± SD from 3 independent experiments. ^*∗*^*p* < 0.05.

These results suggest that loss of miR-326/*βarr-1* locus expression is associated with a “stem-like phenotype.” This notion was further supported by the observation that the expression of miR-326 and *βarr-1* increased in NSCs exposed to differentiation stimuli, for example, retinoic acid (RA) or platelet-derived growth factor (PDGF) (Figures 2(a)–2(d)). Under these conditions NSCs were able to differentiate into distinct lineages as indicated by the increased expression of both neuronal differentiation markers, *β*III-tubulin (*β*III-tub) and NeuN, and astrocytic differentiation markers Gfap and S100 (Figures 2(c) and 2(d)) and downmodulated the expression of stemness related markers Nanog, Oct4, and Sox2 (Figures 2(c) and 2(d)).

### 3.2. Epigenetic Inactivation of miR-326/*β*arr-1 Locus

Next we investigated the mechanism responsible for miR-326/*β*arr-1 locus inactivation in NSCs. There is increasing evidence that epigenetic regulation of stem cells, including “CpG island” methylation, is crucial in the preservation of their stemness by controlling the transcription switch on/off of specific developmental genes [15].

**Figure 3:**
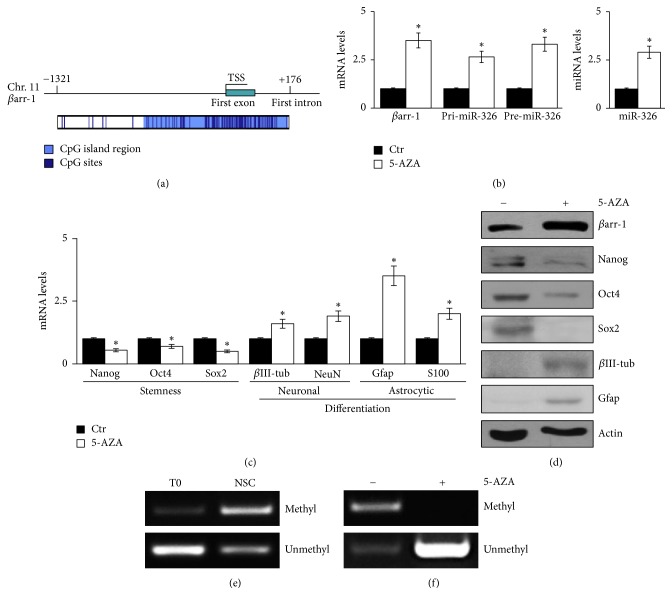
CpG island methylation of miR-326/*β*arr-1 regulatory sequence controls *β*arr-1 and miR-326 expression. (a) Schematic representation of mouse miR-326/*β*arr-1 regulatory regions, highlighting the CpG islands regions (light blue) and the CpG islands sites (blue) derived from DBCAT software. (b) mRNA expression levels of *βarr-1* and precursor forms of* miR-326* and* miR-326* expression levels in NSCs after 4 days of 5-azacytidine (5-AZA) treatment. Data are means ± SD from 3 independent experiments. ^*∗*^*p* < 0.05. ((c)-(d)) mRNA expression levels (c) and Western blot analysis (d) of endogenous *β*arr-1, stemness markers (Nanog, Oct4, and Sox2), and differentiation markers (*β*III-tub and Gfap) after 4 days of 5-azacytidine (5-AZA) treatment in NSCs. Actin as LC. Data are means ± SD from 3 independent experiments. ^*∗*^*p* < 0.05. (e) Methylation specific PCR of the screened region in NSCs compared to the starting bulk population (T0). (f) Methylation specific PCR of the screened region in NSCs after 5-AZA treatment. ((e) and (f)) Data are representative images from 3 independent experiments.

Indeed, the presence of several CpG islands in the region spanning from the first exon to part of the first intron (Figure 3(a) and Supplementary Figure  1: see Supplementary Figure  1 in the Supplementary Material available online at https://doi.org/10.1155/2017/5274171) suggested a DNA methylation-dependent control. Treatment of NSCs with the 5′-azacytidine (5-AZA) demethylating agent induced a significant increase of miR-326, its precursor transcripts, and *β*arr-1 levels (Figure 3(b)) and impaired stemness features while it increased cell differentiation (Figures 3(c) and 3(d)). Using methylation specific PCR we evaluated the methylation status of selected CpG islands. We found that these CpG islands were methylated in NSCs but not in the starting bulk population (T0) (Figure 3(e)). Moreover, when NCSs were treated with 5-AZA, the analyzed CpG islands lost their methylation status (Figure 3(f)), strongly suggesting that the upregulation of *β*arr-1 and miR-326 was due to the demethylation of their promoter region (Figure 3(b)).

These findings highlight that the methylation status of *βarr-1/miR-326* CpG islands is a mechanism to silence their expression in cerebellar NSCs.

### 3.3. *β*arr-1 Negatively Regulates NSCs Self-Renewal via Increasing p27 Nuclear Expression Levels

The above presented data suggested a role of miR-326 and *β*arr-1 in the establishment of a “differentiated phenotype.” miR-326 is known to control several morphogenic signals that sustain stemness, such as the Hedgehog pathway and the Notch pathway [7, 16] and we found it is able to regulate Gli2 and Smo expression also in NSCs (data not shown). Indeed, miR-326 overexpression in NSCs (Figure Supplementary 2A) significantly reduced clonogenicity (Figure Supplementary 2B) and cell viability (Figure Supplementary 2C).

**Figure 4:**
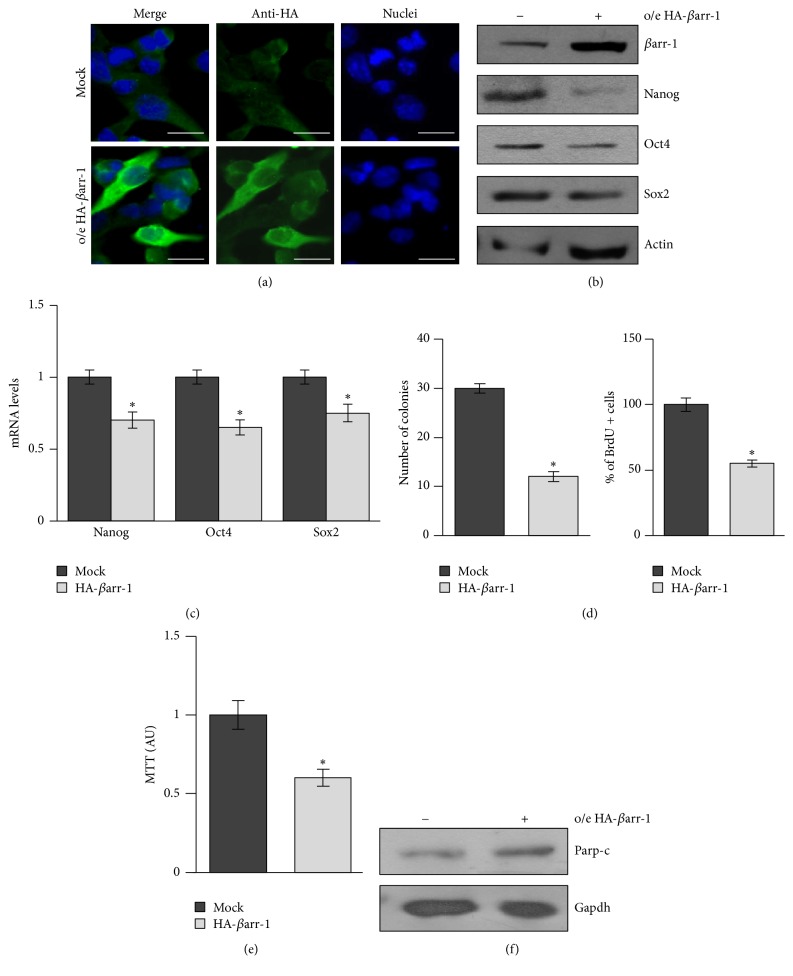
*β*arr-1 overexpression impairs NSCs clonogenicity and proliferation. (a) Representative image of immunofluorescence staining of NSCs after overexpression of HA-*β*arr-1. Nuclei are counterstained with Hoechst. Scale bar: 5 *μ*m. (b) WB analysis of endogenous stemness markers in NSCs after overexpression of HA-*β*arr-1. Loading control: actin. Representative Western blot images from 3 independent experiments. (c) mRNA expression levels of stemness markers in NSCs after overexpression of HA-*β*arr-1. (d) Oncosphere forming assay (number of colonies, left panel) and bromodeoxyuridine (BrdU) uptake (right panel) in NSCs after ectopic expression of HA-*β*arr-1. (e) Cell viability (MTT assay) in NSC after ectopic expression of HA-*β*arr-1. (f) WB analysis of cleaved Parp (Parp-c) in NSCs after overexpression of HA-*β*arr-1. Loading control: Gapdh. Representative Western blot images from 3 independent experiments. ((c), (d), and (e)) Data are means ± SD from 3 independent experiments. ^*∗*^*p* < 0.05.

**Figure 5:**
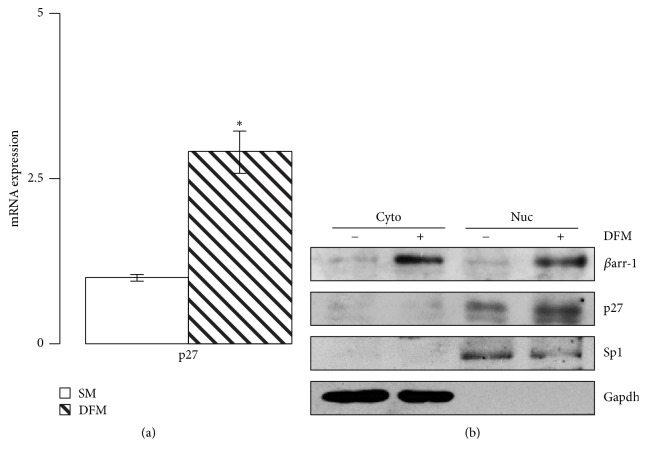
*β*arr-1 controls NSCs proliferation via p27 expression. (a) p27 mRNA expression levels evaluated by qRT-PCR in NSCs grown under stemness conditions (SM) and after differentiation (DFM) for 48 hours. Data are means ± SD from 3 independent experiments. ^*∗*^*p* < 0.05 (DFM versus SM). (b) Western blot showing subcellular localization of endogenous *β*arr-1 and p27 in NSC cultured in SM or DFM for 48 hours. *β*arr-1 and p27 proteins levels were assessed in cytosolic (Cyto) and nuclear (Nuc) fractions. Gapdh and Sp1 protein levels were used as loading controls and markers for purity of Cyto and Nuc fractions, respectively. Representative Western blot images from 3 independent experiments.

**Figure 6:**
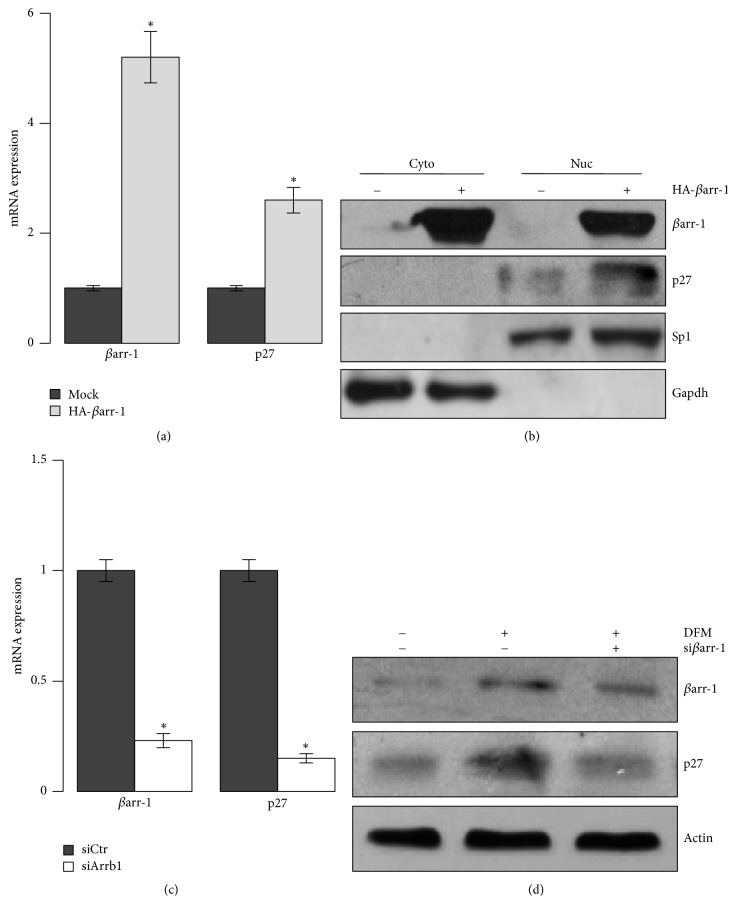
*β*arr-1 controls NSCs proliferation via p27 expression. (a) *β*arr-1 and p27 mRNA expression levels in NSCs transfected with the HA-*β*arr-1 plasmid for 48 hrs. (b) Subcellular localization of *β*arr-1 and p27 proteins in NSCs transfected with the HA-*β*arr-1 plasmid and analyzed 48 hrs after transfection. Proteins are shown in the cytosolic (Cyto) and nuclear (Nuc) fractions. Sp1: nuclear loading control; Gapdh: cytoplasmic loading control. Representative Western blot images from 3 independent experiments. (c) *βarr-1* and* p27* mRNA expression levels in NSCs transfected with control siRNA (siCtr) or *β*arr-1 siRNA (si*β*arr-1) cultured for 24 hrs in DFM. (d) Western blot showing endogenous *β*arr-1 and p27 protein levels in NSCs transfected with control siRNA (siCtr) or *β*arr-1 siRNA (si*β*arr-1) cultured for 24 hrs in DFM. Representative Western blot images from 3 independent experiments. ((a) and (c)) Data are means ± SD from 3 independent experiments. ^*∗*^*p* < 0.05.

On the other hand, previous studies have shown that *βarr-1* functions as a cytoplasm-nucleus shuttling protein that interacts with p300/CBP to activate the transcription of the cyclin-dependent kinase (CDK) inhibitor Cdkn1b/p27kip (p27), a major determinant of cell cycle exit [16]. To gain insight into *βarr*-*1* functions in neuronal cells stemness and differentiation, we overexpressed *βarr-1* (Figures 4(a) and 4(b)) and found a strong impairment of the expression levels of the stemness markers Nanog, Oct4, and Sox2 (Figures 4(b) and 4(c)), impaired clonogenicity together with decreased proliferation rate and cell viability (Figures 4(d) and 4(e)), and increased cell apoptosis (Figure 4(f)), suggesting that *βarr-1* may have a role both in the regulation of stemness features and in the regulation of cell cycle. Indeed, we found that, when shifted to differentiation medium, NSCs increased *βarr-1* level was paralleled by an activation of p27 transcription and the increase of p27 protein in the nucleus as observed in nucleous/cytoplasmic fractionation experiment (Figure 5). Accordingly, p27 mRNA and the nuclear protein cell fraction levels were increased by exogenously expressed *βarr-1* (Figures 6(a) and 6(b)) and decreased by siRNA-mediated abrogation of *βarr-1* expression (Figures 6(c) and 6(d)).

Altogether these results show that *βarr-1* expression inhibits the cell cycle via activation of p27 thus blocking cell stemness features in NSCs.

## 4. Discussion

**Figure 7:**
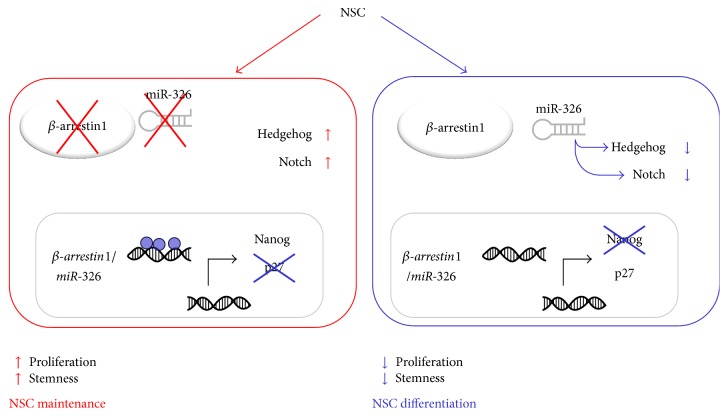
miR-326 and *β*-arrestin1 are epigenetically silenced and control stemness features in cerebellar NSCs. We propose a model in which epigenetic silencing of the intragenic miR-326 and its host gene, *β*-arrestin1, maintains physiological neuronal stemness. In cerebellar NSCs miR-326 and *β*-arrestin1 are epigenetically silenced through hypermethylation of CpG islands on their common promoter, leading to a permissive molecular environment for the expression of prostemness and proproliferative cues. Upon differentiation or demethylating treatment, the promoter of the locus miR-326/*β*-arrestin1 is demethylated and thus the expression levels of miR-326 and *β*-arrestin1 are upregulated. While miR-326 targets molecules belonging to the Hedgehog pathway and the Notch pathway, thus hampering stemness properties, *β*-arrestin1 acts as a cofactor for the transcriptional activation of p27, leading to cell cycle arrest.

In this study we propose a model in which epigenetic silencing of the intragenic miR-326 and its host gene, *βarr-1*, maintains physiological neuronal stemness. While miR-326 and *βarr-1* are highly expressed in differentiated cells where they induce growth arrest, in NSCs they are kept at low levels through CpG hypermethylation. Coherently, modulation of the expression of *βarr-1* through overexpression regulated the differentiation and growth rate of cerebellar NSCs. The reactivation of the locus miR-326/*βarr-1* enhances the cell cycle inhibitor p27 while inhibiting proliferative signalling (e.g., Hh or Notch by miR-326), thus resulting in stem cell differentiation and growth arrest (Figure 7).

These findings suggest a new circuitry composed of miR-326/*β*Arr-1, morphogenic molecules, and cell cycle modifiers which control neural stemness status.

In particular, here we identify *βarr-1* as a new player in the coordinated control of cerebellar NSCs. *βarr-1* was first identified as a gene encoding a scaffold protein that regulates G-protein-coupled receptor (GPCR), through interaction with cytoplasmic proteins linking GPCRs to intracellular signaling pathways [17, 18]. Our observations of the role of *βarr-1* in NSCs are consistent with the growth arrest observed in GCPs where *βarr-1* directly affects gene expression, translocating into the nucleus where it interacts with transcription cofactors at the promoters of the target gene p27 [19].

Consistently, previous studies investigated the role of this CDK-inhibitor in suppressing self-renewal and proliferation while driving differentiation of NSCs [17, 18, 20, 21] and cerebellar progenitors [22, 23]. A further link between p27 and stem/progenitor cell differentiation is provided by the p27-destabilizing effect induced by REST, a repressor of neuronal differentiation, resulting in maintenance of cell proliferation and blockade of neuronal differentiation [24]. In this way, loss of p27 maintains a high turnover of self-renewing cells, by coupling the ability to control both cell cycle and undifferentiated status. Of note, *β*arr-1 overexpression in NSCs affected also cell viability and increased cell apoptosis suggesting that such scaffold adaptor protein could have other possible functions in controlling different mechanisms besides the cell growth arrest.

Our findings underline a coordinated epigenetic mechanism that finely regulates miR-326/*β*arr-1 expression and cell growth in neural stem cells. Indeed, the same promoter drives the expression of *β*arr-1 and miR-326 through an epigenetic control in cerebellar NSC.

The ability of miR-326/*βarr-1* to be epigenetically regulated also links this transcript to the processes preserving the stemness status. Indeed, increasing evidence supports the crucial role of epigenetic regulation of stem cells, including NSCs [25]. Such a regulation includes the mechanisms responsible for the maintenance of repressive chromatin states at specific loci, consisting of CpG islands methylation [26].

Accordingly, in our study miR-326 and *β*arr-1 are among the silenced genes in NSCs, whose regulation contributes to balance the opposing forces of cell growth and differentiation. Indeed the miR-326/*β*arr-1 promoter is characterized by methylated or demethylated CpG islands in NSC or differentiated cells, respectively.

Interestingly, in line with this, miR-326 and *β*arr-1 have been described to be key molecules in multiple sclerosis pathogenesis, being crucial for CD4+ T cell survival and differentiation. Indeed *β*arr-1 enhanced the expression of the protooncogene Bcl2 via the acetylation of histone H4 at the Bcl2 promoter [27] while miR-326 promoted interleukin 17- (IL-17-) producing T helper cells (TH-17) differentiation by targeting Ets-1, a negative regulator of TH-17 differentiation [28]. Of note CD4+ T cells from patients with multiple sclerosis had much higher Arrb1 expression [27] and miR-326 expression was highly correlated with disease severity in patients with multiple sclerosis [28].

Even if no specific studies of *β*arr-1/miR-326 coregulation have been conducted in multiple sclerosis our results together with the abovementioned observations allow speculating that the *β*arr-1/miR-326 transcription unit is regulated in other stem or progenitor cells during differentiation, participating in the control of cell fate, development, and disease.

## 5. Conclusions

We describe in the present work the role and regulation of the locus miR-326/*β*arr-1. miR-326 and its host gene *β*arr-1 represent a novel miRNA/protein network that controls cerebellar NSCs through the modulation of morphogenic signals and cell cycle modifiers, both regulating stemness and suggested to be involved in the maintenance of self-renewal feature of NSCs.

In conclusion, our findings describe a bivalent signal (miRNA and hosting protein encoding gene) converging upon the coordinated inhibition of normal stem cell functions.

## Supplementary Material

Supplementary Figure 1: Detailed report of β-arrestin1 regulatory sequence: the analyzed CpG by methylation specific PCR are evidenced in light blue. The first exon is depicted in blue. Supplementary Figure 2: (a) miR-326 levels in NSC after ectopic expression of miR-326. ^*^P < 0.05. (b) Oncosphere forming assay (number of colonies, left panel) in NSC after ectopic expression of miR-326. ^*^P < 0.05. (c) Cell viability (MTT assay) in NSC after ectopic expression of miR-326. ^*^P < 0.05
