# Supplementary material for: β-Arrestin1/miR-326 Transcription Unit Is Epigenetically Regulated in Neural Stem Cells Where It Controls Stemness and Growth Arrest
Source: Stem Cells Int. 2017 Feb 12;2017:5274171. doi: 10.1155/2017/5274171 (PMC5337365; doi:10.1155/2017/5274171)
Supplement: Supplementary file 1 — Supplementary Figure 1: Detailed report of β-arrestin1 regulatory sequence: the analyzed CpG by methylation specific PCR are evidenced in light blue. The first exon is depicted in blue. Supplementary Figure 2: (a) miR-326 levels in NSC after ectopic expression of miR-326. *P < 0.05. (b) Oncosphere forming assay (number of colonies, left panel) in NSC after ectopic expression of miR-326. *P < 0.05. (c) Cell viability (MTT assay) in NSC after ectopic expression of miR-326. *P < 0.05 [file 5274171.f1.pdf]

Supplementary Figure Legends

Supplementary Figure 1.

Detailed report of β-arrestin1 regulatory sequence: the analyzed CpG by methylation specific PCR are evidenced in light blue. The first exon in depicted in blue.

Supplementary Figure 2.

A. miR-326 levels in NSC after ectopic expression of miR-326. * $p < 0.05$
B. Oncosphere forming assay (number of colonies, left panel) in NSC after ectopic expression of miR-326. * $p < 0.05$
C. Cell viability (MTT assay) in NSC after ectopic expression of miR-326. * $p < 0.05$

Data in A-C are means±SD from 3 independent experiments.

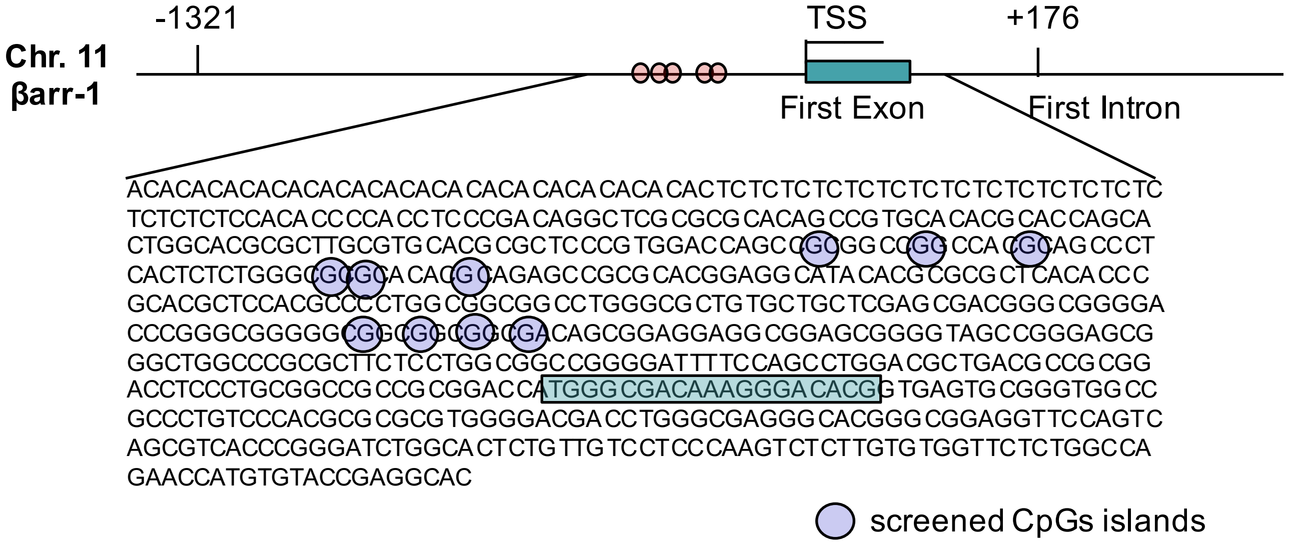

Supplementary Figure 1

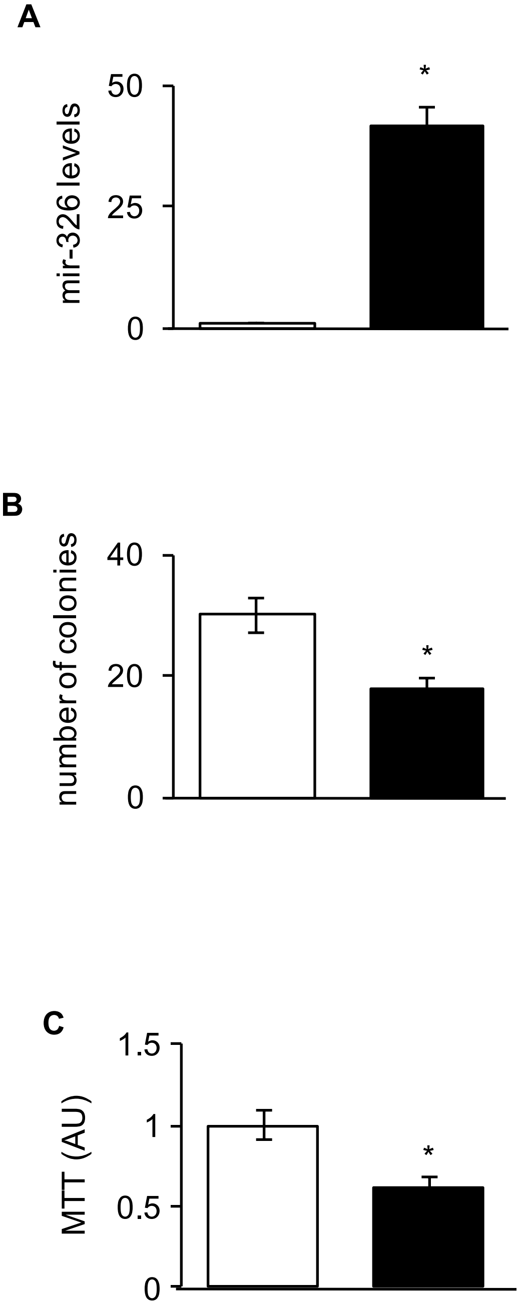

Supplementary Figure 2
